# How Does Learnability of Primary Care Resident Physicians Increase After Seven Months of Using an Electronic Health Record? A Longitudinal Study

**DOI:** 10.2196/humanfactors.4601

**Published:** 2016-02-15

**Authors:** Martina A Clarke, Jeffery L Belden, Min Soon Kim

**Affiliations:** ^1^ Department of Internal Medicine Division of Cardiology University of Nebraska Medical Center Omaha, NE United States; ^2^ Department of Family and Community Medicine University of Missouri Columbia, MO United States; ^3^ Department of Health Management and Informatics University of Missouri Columbia, MO United States; ^4^ Informatics Institute University of Missouri Columbia, MO United States

**Keywords:** primary care, physicians, usability, electronic health records, computerized physician order entry, user-computer interface

## Abstract

**Background:**

Electronic health records (EHRs) with poor usability present steep learning curves for new resident physicians, who are already overwhelmed in learning a new specialty. This may lead to error-prone use of EHRs in medical practice by new resident physicians.

**Objective:**

The study goal was to determine learnability gaps between expert and novice primary care resident physician groups by comparing performance measures when using EHRs.

**Methods:**

We compared performance measures after two rounds of learnability tests (November 12, 2013 to December 19, 2013; February 12, 2014 to April 22, 2014). In Rounds 1 and 2, 10 novice and 6 expert physicians, and 8 novice and 4 expert physicians participated, respectively. Laboratory-based learnability tests using video analyses were conducted to analyze learnability gaps between novice and expert physicians. Physicians completed 19 tasks, using a think-aloud strategy, based on an artificial but typical patient visit note. We used quantitative performance measures (percent task success, time-on-task, mouse activities), a system usability scale (SUS), and qualitative narrative feedback during the participant debriefing session.

**Results:**

There was a 6-percentage-point increase in novice physicians’ task success rate (Round 1: 92%, 95% CI 87-99; Round 2: 98%, 95% CI 95-100) and a 7-percentage-point increase in expert physicians’ task success rate (Round 1: 90%, 95% CI 83-97; Round 2: 97%, 95% CI 93-100); a 10% decrease in novice physicians’ time-on-task (Round 1: 44s, 95% CI 32-62; Round 2: 40s, 95% CI 27-59) and 21% decrease in expert physicians’ time-on-task (Round 1: 39s, 95% CI 29-51; Round 2: 31s, 95% CI 22-42); a 20% decrease in novice physicians mouse clicks (Round 1: 8 clicks, 95% CI 6-13; Round 2: 7 clicks, 95% CI 4-12) and 39% decrease in expert physicians’ mouse clicks (Round 1: 8 clicks, 95% CI 5-11; Round 2: 3 clicks, 95% CI 1-10); a 14% increase in novice mouse movements (Round 1: 9247 pixels, 95% CI 6404-13,353; Round 2: 7991 pixels, 95% CI 5350-11,936) and 14% decrease in expert physicians’ mouse movements (Round 1: 7325 pixels, 95% CI 5237-10,247; Round 2: 6329 pixels, 95% CI 4299-9317). The SUS measure of overall usability demonstrated only minimal change in the novice group (Round 1: 69, high marginal; Round 2: 68, high marginal) and no change in the expert group (74; high marginal for both rounds).

**Conclusions:**

This study found differences in novice and expert physicians’ performance, demonstrating that physicians’ proficiency increased with EHR experience. Our study may serve as a guideline to improve current EHR training programs. Future directions include identifying usability issues faced by physicians when using EHRs, through a more granular task analysis to recognize subtle usability issues that would otherwise be overlooked.

## Introduction

### Physicians’ Electronic Health Records (EHR) Use

Health information technology’s (HIT) functionality in clinical practice is expanding and physicians are increasingly adopting EHRs as a result of the financial incentives guaranteed by Centers for Medicare & Medicaid Services (CMS) [1]. Meaningful Use (MU) is one measure of successful adoption of EHRs as a component of the Health Information Technology for Economic and Clinical Health (HITECH) act proposed by the Office of the National Coordinator for Health Information Technology (ONC) and CMS. EHRs are “records of patient health information generated by visits in any health care delivery setting” [2]. EHRs center on the overall health of a patient beyond clinical data gathered from a single provider, and offer a more comprehensive view of a patient’s care. EHRs are designed for sharing data with other health care providers such as laboratories and specialists; therefore, EHRs contain information from every clinician involved in a patient’s care [3]. In a data brief in 2013, the National Center for Health Statistics (NCHS) reported that 78% of office-based physicians had adopted EHRs in their practice [4]. Presently, EHRs require a large investment of effort for users to become proficient in their use. Resident physicians were selected for this study because those who are not adequately trained in using EHRs may experience a steep learning curve when beginning their residency program [5]. In an effort to maximize physician proficiency with EHRs, hospitals and clinics provide comprehensive EHR training for their resident physicians. However, it is challenging to find sufficient time to train physicians to use new EHR systems [6-9]. Using information technology to manage the process of patient care and to communicate with patients is an essential redesign of clinical practice [10]. Some advantages expressed by EHR users of adopting an EHR consist of the following: increased adherence to guidelines in preventive care, decreased paperwork for providers, and improvement in overall quality and efficiency of patient care [11-13]. Nonetheless, there are possible drawbacks to EHRs: financial burden, mismatch of human and machine workflow models, and productivity loss potentially caused by EHR usability issues [11,12,14-22]. Usability is described as the degree to which software can by employed by users to effectively perform a particular task in a specific content area [23]. EHRs with poor usability may have a negative effect on clinicians’ EHR learning experience. This could lead to increased cognitive load, medical errors, and a decline in quality of patient care [24-29]. Learnability is defined as the extent to which a system permits users to understand how to use it [30]. Learnability deals with the amount of time and effort needed for a user to develop proficiency with a system over time and after multiple use [31]. In the literature, while there are variations in defining usability and learnability [32-34], definitions of learnability are strongly correlated with usability and proficiency [33,35,36]. Allowing physicians to efficiently accomplish clinical tasks within the EHR may ease time constraints experienced by physicians during patient visits.

According to an EHR user satisfaction survey completed in 2012 by 3088 family physicians, approximately 62% of survey respondents were not satisfied with many of the best-known EHR systems, and EHR vendor support and training were the areas with lowest satisfaction ratings [37]. Multiple studies on successful EHR implementations have stressed the usefulness of training in the implementation process [7,9,38-47]. A survey by Aaronson et al [44] concerning EHR use in 219 family practice residency programs indicated that resident physicians’ EHR training may have an impact not only on perceived ease of use of EHR systems, but also on the use of EHR systems in their practices after residency.

### Prior EHR Usability Evaluation Studies

Previous studies have shown the importance of usability evaluation in the EHR adoption and implementation process. Current best practices promote the use of cognitive approaches to examine human-computer interactions in EHR systems [2,48-50]. Khajouei and Jasper performed a systematic review examining the impact of the design aspects of medication systems in computerized physician order entry systems (CPOE) (usually integrated in EHRs) on usability. They found that proper CPOE system design is fundamental to promoting physicians' adoption and diminishing medication errors [51]. Multiple studies have used heuristic evaluation as a method to identify usability issues in health information technology. Chan et al evaluated the usability of a CPOE order set system using heuristic evaluation and discovered 92 unique heuristic violations across 10 heuristic principles [52]. Harrington and Porch investigated an EHR’s usability and identified 14 usability heuristics that were violated 346 times in the intensive care unit clinical documentation [53]. Li et al evaluated clinical decision support with simulated usability testing using a think-aloud protocol, and found that 90% of negative comments from users were concerning navigation and workflow issues [54]. In a study at an urban medical center in New York, Kushniruk et al probed the association between usability tests and training of a commercial EHR system. About 1 month after in-class training, laboratory-based usability testing containing 22 sets of scenario-based tasks was conducted. Usability issues were identified as physicians completed their tasks, leading to numerous areas of potential improvement for system learnability and usability.

### Objective

EHRs with poor usability present steep learning curves for new resident physicians, who are already overwhelmed in learning a new specialty. This may lead to error-prone use of EHRs in medical practice by new resident physicians. Identifying and addressing early barriers in the learning environment can help improve the overall capacity of new physicians and save costs for organizations. The objective of this study was to determine the difference in learnability by comparing changes in performance measures between expert and novice primary care physicians 3 and 7 months after 2 rounds of learnability tests (Round 1: November 12, 2013 to December 19, 2013; round 2: February 12, 2014 to April 22, 2014). We analyzed learnability by addressing 2 specific research questions: (1) Do performance measures of expert and novice physicians improve after 3 and 7 months of EHR experience? and (2) Does the learnability gap between novice and expert physician groups change after 7 months of EHR experience?

## Methods

### Study Design

To determine learnability gaps between expert and novice physicians when using EHRs, data were collected through learnability testing using Morae video analysis software (TechSmith). Twelve family medicine and 4 internal medicine resident physicians performed 19 artificial, scenario-based tasks in a laboratory setting. Four types of quantitative performance measures, a system usability scale (SUS), a survey instrument [55], and a qualitative debriefing session with participants were employed. This study was approved by the University of Missouri Health Sciences Institutional Review Board.

### Organizational Setting

This study took place at the University of Missouri Health System (UMHS), which is a 536-bed, tertiary-care academic medical hospital located in Columbia, Missouri. In 2012, UMHS had approximately 553,300 clinic visits and employed more than 70 primary care physicians. The Department of Family and Community Medicine (FCM) runs 6 clinics, while the Department of Internal Medicine (IM) oversees 2 primary care clinics [56]. The Healthcare Information and Management Systems Society (HIMSS), a non-profit organization that scores how effectively hospitals employ electronic medical record (EMR) applications, assigned UMHS a rating of Stage 7 with respect to the EMR Adoption Model [57]. In other words, UMHS has adopted electronic patient charts, examined clinical data through data warehousing, and shares health information electronically with authorized health care bodies [58]. The CPOE within the EHR permits physicians to safely and electronically access and place lab and medication orders for patients, and transfer orders directly to departments that are responsible for implementing requests. UMHS’ EHR database comprises all data from the university’s hospitals and clinics. University of Missouri Health Care has been using a mature EHR system since 2003 from the same vendor. New users of the EHR receive 4 to 8 hours of training and also have drop-in access (or can book an appointment) to an EHR Help Room to receive help or further training. Supplemental online training materials such as documents, videos, and self-paced tutorials are also available. When new features are included in the EHR, illustrated instructions and explanations become available.

### Participants

There is presently no evidence-based approach to measure a user’s EHR experience; therefore, novice and expert physicians were distinguished based on clinical training level and number of years using the EHR. This decision was based on a discussion with an experienced physician champion (JLB). This study will examine and confirm if after 1 year of EHR use, resident physicians have gained sufficient skills to be considered an expert [59]. Thus, 10 first-year resident physicians were grouped as novice users and 6 second and third-year resident physicians were grouped as expert users. Both FCM and IM run 3-year residency programs. A convenience sampling method was used when choosing participants [60]. UMHS FCM and IM physicians were selected for the sample because, as primary care residents, they have equivalent clinical roles and duties. Based on a review of the literature, a sample of 15 to 20 participants was judged suitable for exploratory usability studies to identify major problems to correct in a product development cycle [61-63]. However, we observed data saturation in terms of usability issues at 5 participants. Participation was voluntary and subjects were compensated US $20 for their involvement in the project.

In Round 1, 10 novice physicians and 6 expert physicians participated in the study. Out of the 10 novice physicians in Round 1, 7 were from family medicine and 3 from internal medicine. Of the 10 novice physicians, 6 (60%) were male, 8 (80%) identified their race as white, 1 (10%) identified as Asian, and 1 (10%) identified as both Asian and white. The age of novice physicians ranged from 27 to 31 and the mean age was 28 years. In Round 1, 4 (40%) novice physicians had no experience with an EHR other than the one at UMHS, 2 (20%) had less than 3 months of experience, 1 (10%) had 7 months to 1 year of experience, and 3 (30%) had over 2 years of experience with an EHR other than the one at UMHS. In this study, 5 family medicine and 1 internal medicine expert physicians participated in the study. Of the 6 expert physicians, 5 (83%) were female and all (100%) identified their race as white. In this study, 2 did not provide information on their date of birth and EHR experience and were not included in the calculation of age range, mean age, and EHR experience. The age of expert physicians ranged from 30 to 33 and the mean age was 31 years. In this study, 1 (17%) expert physician had no experience with an EHR other than the one at UMHS, 1 (17%) had 7 months to 1 year of experience, and 2 (33%) had over 2 years of experience with an EHR other than the one at UMHS.

Of the 8 novice physicians and 4 expert physicians who participated in Round 1 also participated in Round 2 of the study. A total of 2 novice and 2 expert physicians who participated in Round 1 declined participation in Round 2. Conducting 2 rounds of data collection was a major strength of this study, because it allowed us to measure valid learnability. Out of the 8 novice physicians in Round 2, 5 were from family medicine and 3 from internal medicine. Of the 8 novice physicians, 5 (63%) were male, 8 (75%) identified their race as white, 1 (13%) identified as Asian, and 1 (13%) identified as both Asian and white. The age of novice physicians ranged from 27 to 30 and the mean age was 28 years. In Round 2, 3 (38%) novice physicians had no experience with an EHR other than the one at UMHS, 2 (25%) had less than 3 months of experience, 1 (13%) had 7 months to 1 year of experience, and 2 (25%) had over 2 years of experience with an EHR other than the one at UMHS. Four family medicine expert physicians participated in the study. All 4 (100%) were female and all (100%) identified their race as white. The age of expert physicians ranged from 30 to 33 and the mean age was 31 years. In this study, 1 (25%) expert physician had no experience with an EHR other than the one at UMHS, 1 (25%) had 7 months to 1 year of experience, and 2 (50%) had over 2 years of experience with an EHR other than the one at UMHS. Because of the small sample size, we did not attempt to control for age or gender.

### Scenario and Tasks

Two sets of artificial but realistic scenario-based tasks were used in the study. The tasks were created based on discussion with an experienced physician champion (JLB) and 2 chief resident physicians from both participating departments (FCM, IM). When completing Round 1 of the learnability test, resident physicians were given a scenario for a “scheduled follow-up visit after a hospitalization for pneumonia.” When completing Round 2 of the learnability test, resident physicians were given a scenario for a “scheduled follow-up visit after a hospitalization for heart failure.” While different, these 2 scenarios were equivalent in difficulty, workflow, and functionalities used. These scenarios were employed to assess physicians’ use of the EHR with realistic inpatient and outpatient information. We included 19 tasks that are generally completed by both novice and expert primary care physicians. These tasks also met 2014 EHR certification criteria 45 CFR 170.314 for meaningful use (MU) Stage 2 [31]. The alphanumeric code located beside each task corresponds to the EHR certification criteria that satisfies meaningful use Stage 2 objectives. In order to measure learnability more effectively, we confirmed that the tasks were also practiced during EHR training required of resident physicians at the commencement of their residency. The tasks had clear objectives that physicians were able to follow without needless clinical cognitive load or ambiguity, which would have deviated from the study aim. The tasks were as follows:

1. Start a new note (§170.314[e][2]).

2. Include visit information (§170.314[e][2]).

3. Include chief complaint (§170.314[e][2]).

4. Include history of present illness (§170.314[e][2]).

5. Review current medications contained in the note (§170.314[a][6]).

6. Review problem list contained in the note (§170.314[a][5]).

7. Document new medication allergy (§170.314[a][7]).

8. Include review of systems (§170.314[e][2]).

9. Include family history (§170.314[a][13]).

10. Include physical exam (§170.314[a][4] and §170.314[e][2]).

11. Include last comprehensive metabolic panel (CMP) (§170.314[b][5]).

12. Save the note.

13. Include diagnosis (§170.314[a][5]).

14. Place order for chest X-ray (§170.314[a][1] and §170.314[e][2]).

15. Place order for basic metabolic panel (BMP) (§170.314[a][1] and §170.314[e][2].

16. Change a medication (§170.314[a][1] and §170.314[a][6].

17. Add a medication to your favorites list (§170.314[a][1].

18. Renew one of the existing medications (§170.314[a][1] and §170.314[a][6].

19. Sign the note.

### Performance Measures

Learnability was evaluated using 4 quantitative performance measures. *Percent task success* was the percentage of subtasks that participants successfully completed without error. *Time-on-task* calculated how long in seconds it took each participant to complete each task. Calculation began when a participant clicked on the “start task” button and ended when the “end task” button was clicked. *Mouse clicks* computed the number of times the participant clicked on the mouse when completing a given task. *Mouse movement* calculated in pixels the distance of the navigation path by the mouse to complete a given task.

For percent task success rate, a higher value usually signifies better performance, representing participants’ skill with the system. For time-on-task, mouse clicks, and mouse movements, a higher value usually indicates poorer performance [62,64,65]. As such, higher values may indicate that the participant encountered complications while using the system.

### System Usability Scale

After testing, participants were asked to complete the System Usability Scale (SUS) to supplement the performance measures. The SUS is a 10-item survey measured on a Likert scale that provides fairly robust measures of subjective usability and is a widely used, validated instrument in HIT evaluation [31,55,66]. The SUS produces a single score (ranging from 0 to 100, with 100 being a perfect score [55]) that represents a composite measure of the overall usability of the system under examination. A score of 0 to 50 is considered not acceptable, 50 to 62 is low marginal, 63 to 70 is high marginal, and 70 to 100 is acceptable.

### Data Collection

Two rounds of data collection were scheduled to measure learnability by comparing whether participants’ performance measures (task success, time-on-task, mouse clicks, and mouse movements) improved and if participants experienced fewer usability issues with longer exposure to the system. Learnability pertains to the amount of time and effort needed for a user to develop proficiency with a system over time and after multiple use [31]. The 2 groups (novice and expert physicians) were essential for our comparison, because experts’ measures were used to examine novices’ improvements toward becoming an expert. Round 1 learnability data were collected between November 12, 2013 and December 19, 2013 and Round 2 data were collected between February 12, 2014 and April 22, 2014. Round 1 data collection began 3 months after novice (Year 1) resident physicians completed their initial mandatory EHR training at UMHS. Resident physicians were invited to complete Round 2 approximately 3 months after the date they completed Round 1. Learnability testing was completed in approximately 20 minutes and conducted on a 15-inch laptop using Windows 7 operating system. To preserve consistency and reduce undesirable interruptions, the participant and facilitator were the only 2 individuals in the conference room. At the beginning of the session, participants were advised that their participation in the study was voluntary and they had the right to end the session at any time. Participants were provided with a binder that contained instructions on how to complete the task before the test began. Tasks were displayed at the top of the display as the test progressed. A think-aloud strategy was used throughout the session and audio, video, on-screen activity, and inputs from the keyboard and mouse were recorded using a Morae Recorder [67,68]. We prompted participants to talk aloud and describe what they were doing while completing the tasks. Participants completed the tasks without the assistance of the facilitator who would only intervene if there were any technical difficulties. However, there were none and the facilitator did not have to intervene. After participants completed the tasks, they completed the SUS and demographic survey. The test session concluded with a debriefing session during which participants were asked to comment on the specific tasks they found difficult. Interesting observations detected by the facilitator were discussed as well.

### Data Analysis

We confirmed there were no EHR interface changes between data collection in Rounds 1 and 2 that may have influenced the study and tasks. The recorded sessions were examined using Morae Manager, a video analysis software program that was used to calculate performance measures using markers to identify difficulties and errors the participants encountered. Video analysis took approximately 1.5 hours for each 20-minute recorded session. The first step in the analysis was to review the recorded sessions and label any tasks that were unmarked during data collection. The second step was to divide each of the 19 tasks into smaller tasks to determine the task success rate and identify subtle usability challenges that we may have otherwise failed to notice. Geometric means were calculated for the performance measures with confidence intervals at 95% [69]. Performance measures have a strong tendency to be positively skewed, so geometric means were used because they provide the most accurate measure for sample sizes less than 25 [70]. The learnability comparison was a *between* comparison of 2 *within* comparisons. Therefore, we measured the difference between the novice and expert resident physician groups and the difference within novice and expert physician groups, 3 and 7 months after EHR training. Comparisons of learnability between the 2 groups were *between* comparisons. Time-on-task, mouse clicks, and mouse movements were measured while users interacted with the EHR system and performance measures were calculated automatically by the Morae Manager usability analysis software program. Percent task success was calculated by creating subtasks out of each task and then identifying each subtask the physician completed successfully. For example, for Task 8 (Include review of systems) the subtasks created to calculate the task success rate were the following: (1) go to review of systems, (2) add “no chills,” (3) add “no fever,” (4) add “fatigue,” (5) add “decreased activity,” (6) add “dry mouth,” (7) add “no dyspnea,” and (8) add “no edema.”

## Results

### Percent Task Success Rate

Geometric mean values of percent task success rates were compared between the 2 physician groups across 2 rounds (Table 2) [69]. There was a 6-percentage-point increase in the novice physician group’s percent task success rate between Round 1 (92%, 95% CI 87%-99%) and Round 2 (98%, 95% CI 95%-100%). Similarly, expert physicians had a 7-percentage-point increase in percent task success rate between Round 1 (90%, 95% CI 83%-97%) and Round 2 (97%, 95% CI 93%-100%). When mean task success rates were compared between the physician groups, the novice physician group had a higher task success rate than the expert physician group did for both rounds.

**Table 2:** Geometric mean values of performance measures for novice and expert physicians across two rounds.

Performance Measures	Round 1 Novice	Round 2 Novice	Round 1 Expert	Round 2 Expert
Task Success	92%	98%	90%	97%
Time-on-Task	44	40	39	31
Mouse Clicks	8	7	8	5
Mouse Movements	9247	7992	7325	6329

In Round 1, the novice physician group achieved a higher success rate than expert physicians for 7 tasks (2, 8, 11, 13, and 15-17), the same success rate for 7 tasks (1, 3-6, 9, and 19), and a lower success rate for 5 tasks (7, 10, 12, 14, and 18). In Round 2, the novice physician group achieved a higher success rate for 3 tasks (8, 9, and 14), the same success rate for 15 tasks (1-7, 10-13, and 16-19), and a lower success rate for Task 15.

Both novice (6%) and expert physician groups (2%) had equally low task success for Task 7 (Add a medication to your favorites list) in Round 1. However, in Round 2 all physicians in both groups successfully completed Task 7 (100%).

### Time-on-Task

Geometric mean values of time-on-task (TOT) were compared between the 2 physician groups across the 2 rounds (Table 2). There was a 10% decrease in novice physicians’ time-on-task between Round 1 (44s, 95% CI 32-62) and Round 2 (40s, 95% CI 27-59). There was a 21% decrease in the expert physician group’s time-on-task between Round 1 (39s, 95% CI 29-51) and Round 2 (31s, 95% CI 22-42). When time-on-task was compared between the physician groups, the overall novice physician group spent more time compared to the expert physician group for both rounds.

In Round 1, the novice physician group spent less time than expert physicians completing 4 out of 19 tasks (5, 11, 12, and 13), the same amount of time completing Task 17, and more time completing 14 tasks (1-4, 6-10, 14-16, 18, and 19). In Round 2, the novice physician group spent less time completing 4 out of 19 tasks (2, 6, 11, and 12), the same time completing Task 18, and more time completing 14 tasks (1, 3-5, 7-10, 13-17, and 19).

In Round 1, both physician groups had the longest time spent on Task 7 (Document new medication allergy). However, in Round 2, time on Task 7 decreased by 52% for the expert physician group (87s to 50s) and 29% for the novice physician group (133s to 95s).

### Mouse Clicks

Geometric mean values of mouse clicks were compared between the 2 physician groups across the 2 rounds (Table 2). There was a 20% decrease in the novice physician group’s mouse clicks between Round 1 (8 clicks, 95% CI 6-13) and Round 2 (7 clicks, 95% CI 4-12). Similarly, there was a 39% decrease in the expert physician group’s mouse clicks between Round 1 (8 clicks, 95% CI 5-11) and Round 2 (5 clicks, 95% CI 1-10). When mouse clicks were compared between the physician groups, the novice physician group completed tasks with slightly more mouse clicks than expert physicians did in both rounds.

In Round 1, the novice physician group achieved lower mouse clicks than the expert physician group for 7 tasks (4, 6, 8, 11, 13, 17, and 19), higher mouse clicks for 9 tasks (1, 5, 7, 9 10, 12, and 14 – 16), and a comparable number of clicks for 3 tasks (2, 3, and 18). In Round 2, novice physicians used less mouse clicks when completing 6 tasks (8, 10, 11, 13, 18 and 19), the same number of clicks when completing 5 tasks (4-6, 12, and 15), and more clicks completing 8 tasks (1-3, 7, 9, 14, 16, and 17).

In Round 1, both novice and expert physicians had the highest number of mouse clicks out of all tasks when completing Task 7 (Add a medication to your favorites list). However, in Round 2, the task with the highest number of mouse clicks by expert physicians changed from Task 7 to Task 15 (Place order for basic metabolic panel [BMP]) and novice physicians had the highest mouse clicks when completing Task 14 (Place order for chest X-ray) in Round 2, compared to Task 7 in Round 1.

### Mouse Movements

Geometric mean values of mouse movements (the length of the navigation path to complete a given task) were compared between the 2 physician groups across the 2 rounds. There was a 14% increase in novice physicians’ mouse movements between Round 1 (9247 pixels, 95% CI 6404-13,353) and Round 2 (7992 pixels, 95% CI 5350-11,936). There was also a 14% decrease in expert physicians’ mouse movements between Round 1 (7325 pixels, 95% CI 5237-10,247]) and Round 2 (6329 pixels, 95% CI 4299-9317). When mouse movements were compared between the physician groups, the novice physician group showed slightly longer mouse movements than expert physicians did across the 19 tasks in both rounds.

In Round 1, the novice physicians showed longer mouse movements for 15 of 19 tasks (1-4, 6-12, 14-16, and 18), and shorter mouse movements for 4 tasks (5, 13, 17, and 19). In Round 2, novice physicians used shorter mouse movements in completing 8 out of 19 tasks (2, 4, 6, 11-13, 18, and 19) and used longer movements completing 11 tasks (1, 3, 5, 7-10, and 14-17).

In Round 1, novice physicians had the longest mouse movements out of all tasks when completing Task 7 (Add a medication to your favorites list) and expert physicians had the longest mouse movements when completing Task 13 (Include diagnosis). In Round 2, the task with the longest mouse movements by novice physicians was Task 14 (Place order for chest X-ray) compared to Task 7 in Round 1 and expert physicians had the longest mouse movements when completing Task 15 (Place order for basic metabolic panel [BMP]).

### System Usability Scale

In Round 1, 5 out of 6 expert physicians and all 10 novice physicians completed the SUS. In Round 2, all 4 expert physicians and all 9 novice physicians completed the SUS. The SUS illustrated that novice physicians ranked the system’s usability at a mean of 69 (high marginal) in Round 1 compared to 68 (high marginal) in Round 2. Experts rated the system’s usability at a mean of 74 (acceptable) in both rounds. A novice physician and 2 expert physicians had a score of 50 (not acceptable) or below. These results may indicate that expert users who have achieved a certain level of proficiency may be more confident using the EHR than novice users. A debriefing session confirmed the overall learnability test experience but did not reveal specific learnability issues. After analyzing the recording, however, it was clear that physicians encountered some difficulties when completing the tasks.

### Usability Themes

Because of space limitations, a second manuscript is in preparation with a full review of the usability themes. Sub-task analysis was instrumental in identifying multiple usability concerns. We identified 31 common and 4 unique usability issues between the 2 physician groups across 2 rounds. Themes were created by analyzing and combining usability issues to form an overarching theme [71]. Five themes emerged during analysis: 6 usability issues were related to inconsistencies, 9 issues concerning user interface issues, 6 issues in relation to structured data issues, 7 ambiguous terminology issues, and 6 issues in regards to workarounds. An example of an inconsistency issue was illogical ordering of lists in Task 17 (Add a medication to your favorites list), such that the medication list could not be sorted alphabetically when imported into a patient’s visit note. This may frustrate physicians when they cannot discern how to sort the medication list. An example of a user interface issue was the long note template list physicians had to navigate when they completed Task 1 (Start a new note). A lengthy list of different templates was chosen from when creating a note and the templates were not specialty specific, such that searching through the template list and choosing a desired template was time consuming and caused extra cognitive load. An example of a structured data issue was a lack of distinction between columns in Task 9 (Include Family History). In this task, the blue or white columns (indicating negative vs positive findings) for family members were unlabeled, such that physicians were unsure how to mark a family history item “positive.” An example of an ambiguous terminology issue was multiple fields having the same functionality. When completing Tasks 14 and 15, there was no clear difference between the drop-down menu labeled “Requested Start Date,” the drop-down menu labeled “Requested Time Frame,” and the radio button labeled “Future Order.” This could cause future lab tests not to be ordered properly, such that lab tests may not be completed at the right time and patients may have to get the test redone, which adds additional cost for the patient. An example of a workaround was unawareness of functions. When completing Task 13 (Include diagnosis), physicians were not able to move “hypertension” from the problem list to the current diagnosis list, so they re-added “hypertension” as a new problem, which took additional time.

## Discussion

### Principal Findings

Our findings show that there were mixed changes in performance measures and expert physicians were more proficient than novice physicians on all four performance measures.

### Relation to Prior Studies

In our study, differences were found between expert and novice physicians’ performance measures across Round 1 and Round 2. A study by Kjeldskov, Skov, and Stage [72] identifying usability problems encountered by novice and expert nurses examined whether or not usability issues disappeared over time. In this study, 7 nurses completed 14 and 30 hours of training prior to the first evaluation that included 7 tasks and subtasks centered on the core purpose of the system. The same nurses completed the same 7 tasks after 15 months of daily use of the system. All expert subjects solved all 7 tasks either completely or partially while only 2 novice subjects solved all tasks (*P*=.01). No statistically significant difference between novice and expert nurses was found when considering only completely solved tasks (*P*=.08). Our study did not report *P* values due to the small sample size; however, we observed overall improvement in performance measures for both novice and expert physician groups across 2 rounds. The contradictory results from this study and the study by Kjeldskov, Skov, and Stage, suggest that further research is necessary to draw more definite conclusions about task success between novice and expert physicians.

Alternatively, a study by Lewis et al measured performance of novice health sciences students and a predictive model of skilled human performance when performing EHR tasks using a touchscreen. Novice participants were adults with no prior experience using an EHR touchscreen interface using CogTool. CogTool is an open-source user-interface prototyping tool that uses a human performance model to automatically evaluate how efficiently a skilled user can complete a task. Participants completed 31 tasks commonly performed by nurses and patient registration clerks in an Anti-Retroviral Therapy clinic. The mean novice performance time for all tasks was significantly slower than predictions of skilled use (*P*<.00) [73]. Although novice EHR users completed touchscreen tasks slower than a skilled user, they were able to execute some tasks at a skilled level within the first hour of system use. Our study also found novice physicians completing tasks slower than expert physicians, although they decreased their time-on-task by 10% in Round 2. However, our study is different from Lewis et al in that we used human expert physicians instead of a predictive model, which gives a more realistic comparison between novice and expert users. The common findings between this study and those of Lewis et al suggest that physicians become efficient as EHR experience increases, in relation to task completion time, because physicians may become familiar with the system.

Physicians’ perceptions of the usability of a system may have relations to learnability; that is, physicians may find the system more user-friendly (usability) if the amount of time and effort needed to develop proficiency with the system is shorter (learnability). In our study, the SUS, which measures overall usability, illustrated that there was only a slight change in novice (Round 1: 69 [high marginal], Round 2: 68 [high marginal]) and expert (Round 1: 74 [high marginal], Round 2: 74 [high marginal]) physicians’ rankings of the system’s usability. In a study by Haarbrandt et al, primary care providers gave a SUS rating of 70.7 (marginally acceptable) when asked about their perception of a health information exchange system, which was similar to the physicians’ scores in our study. Expert and novice participants found the graphical user interface easy to use; however, they only rated the system as acceptable [74]. Kim et al [62] measured usability gaps in emergency department (ED) nurses, and found that novice ED nurses were not satisfied with their system (43 [unacceptable] to 55 [low marginal]) in comparison to expert nurses who were satisfied (75 to 81 [good to excellent]), which was different from our study’s result. The varying SUS scores from the studies mentioned suggest that physicians with more experience using an EHR are more likely to give the system higher SUS scores. Contrary to the assumption that SUS produce reliable scores, there are mixed results that SUS scores clearly associate with performance measures. For example, Kim et al showed very low correlations between performance measures and SUS Scores, indicating that care needs to be taken when interpreting usability data and comprehensive rather than single measures are necessary.

### Study Limitations

This study had several limitations in terms of the methodology. First, it involved a small sample of physicians; therefore, the sample size may not have been sufficient to obtain statistical significance when reporting quantitative results of learnability. However, the sample size was sufficient when identifying usability issues experienced by participants when interacting with the EHR system. This study was conducted at a health care institution where only 1 EHR system was used and may not be representative of all primary care practice. As such, the study’s findings may have limited generalizability to other ambulatory clinic settings, due to different types of EHR applications and physician practice settings. However, the EHR platform employed in this study is one of the top commercial products with significant market share. Based on data from Office of the National Coordinator for Health Information Technology, Cerner was reported as the primary EHR Vendor by 20% of hospitals participating in the CMS EHR incentive programs, making it the second most implemented EHR in hospitals [75]. Second, a limited number of clinical tasks were used in the learnability test and may not have encompassed other tasks completed by physicians in other clinical scenarios. However, these tasks included realistic inpatient and outpatient tasks that resident physicians would usually complete in a clinical scenario. Third, this study was conducted in a laboratory setting, which did not take into account common distractions physicians may experience during a clinical encounter. Nonetheless, laboratory-based learnability tests allow for flexibility in questioning and give room for more in-depth probing. Direct observation in laboratory learnability testing also allows for interaction between participant and facilitator. Although this study contained some methodological limitations, we believe it to be a well-controlled study that used a rigorous evaluation method with validated performance measures that are widely accepted in HIT evaluation. In addition, the clear instructions allowed physician participants to complete the required tasks without excessive cognitive load.

### Conclusion

Overall, this study identified varying degrees of learnability gaps between expert and novice physician groups that may impede the use of EHRs. Our results suggest that longer experience with an EHR may not be equivalent to being an expert or proficient in its use. The physicians’ interactions with the EHR can be communicated to EHR vendors, to assist in improving the user interface for effective use by physicians. This study may also assist in the design of EHR education and training programs by highlighting the areas (ie, tasks and related features and functionalities) of difficulty that resident physicians face. Resident physicians in primary care are offered extensive EHR training by their institutions. However, it is a great challenge for busy physicians to find time for training. Furthermore, it is an arduous task attempting to meet the needs of users and provide hands-on, on-site support [7], and evidence-based guidelines for training resident physicians effectively on how to use EHRs for patient care are scarce [76]. Thus, our study may also serve as a guideline to potentially improve EHR training programs, which may increase physicians’ performance, by improving competency when using the system.

## Abbreviations

CMS: Centers for Medicare & Medicaid Services
CPOE: computerized physician order entry systems
ED: emergency department
EHRs: electronic health records
FCM: Department of Family and Community Medicine
HIMSS: Healthcare Information and Management Systems Society
HIT: health information technology
HITECH: Health Information Technology for Economic and Clinical Health (act)
IM: Department of Internal Medicine
MU: meaningful use
NCHS: National Center for Health Statistics
ONC: Office of the National Coordinator for Health Information Technology
SUS: system usability scale
TOT: time-on-task
UMHS: University of Missouri Health System
